# Disposable Amperometric Label-Free Immunosensor on Chitosan–Graphene-Modified Patterned ITO Electrodes for Prostate Specific Antigen

**DOI:** 10.3390/molecules27185895

**Published:** 2022-09-11

**Authors:** Liang Yan, Chaoyan Zhang, Fengna Xi

**Affiliations:** 1 Shanxi Bethune Hospital, Shanxi Academy of Medical Sciences, Tongji Shanxi Hospital, Third Hospital of Shanxi Medical University, Taiyuan 030032, China; 2 Tongji Hospital, Tongji Medical College, Huazhong University of Science and Technology, Wuhan 430030, China; 3 Department of Chemistry, Zhejiang Sci-Tech University, Hangzhou 310018, China

**Keywords:** amperometric immunosensor, graphene composite, chitosan, disposable electrode, prostate-specific antigen

## Abstract

A facile and highly sensitive determination of prostate-specific antigen (PSA) is of great significance for the early diagnosis, monitoring and prognosis of prostate cancer. In this work, a disposable and label-free electrochemical immunosensing platform was demonstrated based on chitosan–graphene-modified indium tin oxide (ITO) electrode, which enables sensitive amperometric determination of PSA. Chitosan (CS) modified reduced graphene oxide (rGO) nanocomposite (CS–rGO) was easily synthesized by the chemical reduction of graphene oxide (GO) using CS as a dispersant and biofunctionalizing agent. When CS–rGO was modified on the patterned ITO, CS offered high biocompatibility and reactive groups for the immobilization of recognition antibodies and rGO acted as a transduction element and enhancer to improve the electronic conductivity and stability of the CS–rGO composite film. The affinity-based biosensing interface was constructed by covalent immobilization of a specific polyclonal anti-PSA antibody (Ab) on the amino-enriched electrode surface via a facile glutaraldehyde (GA) cross-linking method, which was followed by the use of bovine serum albumin to block the non-specific sites. The immunosensor allowed the detection of PSA in a wide range from 1 to 5 ng mL^−1^ with a low limit of detection of 0.8 pg mL^−1^. This sensor also exhibited high selectivity, reproducibility, and good storage stability. The application of the prepared immunosensor was successfully validated by measuring PSA in spiked human serum samples.

## 1. Introduction

Prostate cancer is the most common malignant tumor of the male genitourinary system, and its fatality rate ranks second to cancer [1,2]. Early diagnosis and treatment of prostate cancer is key to improving survival. Prostate-specific antigen (PSA) is a single-chain glycoprotein secreted by prostatic alveolar and ductal epithelial cells. Under normal physiological conditions, only trace amount of PSA is present in serum (<4.0 ng mL^−1^) [3]. When a tumor occurs, the normal duct structure is destroyed, causing an increase in concentration. Therefore, PSA is a highly sensitive and specific biomarker for prostate cancer, [4,5] and its detection in serum is of great significance for the early diagnosis, monitoring treatment and prognosis of prostate cancer.

Until now, conventional detection methods for serum PSA have included radioimmunoassay, enzyme-linked immunoassay (ELISA) and chemiluminescence immunoassay [6,7,8]. Among these, radioimmunoassay has high sensitivity, accuracy and precision, but the reagents cause radioactive contamination, and the half-life of the marker is short. The operational steps of ELISA are relatively complicated, and its sensitivity needs to be improved. Although the chemiluminescence immunoassay can achieve fully automated detection and high sensitivity, the luminescence intensity is greatly affected by environmental factors, which causes the working curve to drift over time. Recently, a series of highly sensitive detection methods has been developed to detect PSA, such as electrochemiluminescence (ECL), surface-enhanced Raman spectroscopy, colorimetric analysis, photoelectrochemical immunoassay [9,10,11,12]. However, most of these methods are time-consuming and require expensive equipment, professional operators, and complex processing. Compared with other detection strategies such as optical techniques, electrochemical detection has received extensive attention for its fast detection speed, simple instrumentation, ease of integration, and potential for miniaturization [13,14,15,16]. The development of simple and low-cost electrochemical immunosensors for fast, highly sensitive detection of PSA is highly desirable.

Electrodes are critical to the performance of electrochemical sensors, but traditional noble-metal (e.g., gold and platinum) [17] and glassy carbon electrodes [18,19,20,21] are expensive and have complicated pretreatment procedures such as grinding and polishing. In addition, the electrode surface is easily contaminated, leading to decreased detection sensitivity and accuracy. Compared with conventional electrodes, the outstanding advantage of a disposable electrode is the avoidance of surface contamination [22,23,24]. Indium tin oxide (also tin-doped indium oxide) (ITO) is currently the most widely studied and used transparent electrode material [25,26,27,28]. ITO is a doped n-type semiconductor material: high-valent Sn^4+^ is doped into the In_2_O_3_ material. Since Sn^4+^ is similar to In^3+^ in radius, Sn^4+^ replaces part of In^3+^, thereby generating free electrons. At the same time, the In_2_O_3_ material does not form an ideal stoichiometric ratio during preparation, and part of the O_2_^−^ is separated from the original lattice position, which also leads to the existence of a large number of free electrons, resulting in excellent electrical conductivity. Moreover, ITO has good chemical stability. Up to now, magnetron sputtering, electron-beam evaporation, chemical vapor deposition, and sol-gel technology can directly prepare large ITO film areas of high quality and low cost on substrates such as glass, polyethylene terephthalate (PET) and polyimide (PI). Thus, ITO is an ideal disposable electrode owing to the advantages of a wide potential window, stable electrochemical performance, easy patterning, low cost, and mass production.

An immunoassay usually has two detection modes. One is based on labeling [29]. This technique usually forms a sandwich structure containing a label and often requires two kinds of antibodies and several steps of incubation and washing. Consequently, it suffers from high cost, long detection time and complex operation. In recent years, a label-free immunoassay based on the quantitative analysis of targets by signal changes caused by specific antigen–antibody binding has attracted extensive attention [30,31]. For example, the electrochemical label-free immunoassay measures the change in the electrochemical signal of the probe before and after formation of the antigen–antibody complex on the electrode-supported immunorecognitive interface [32,33]. Compared with an immunoassay based on labeling, a label-free immunoassay has the advantages of high speed, low cost and high simplicity.

In this work, a disposable, label-free amperometric immunosensor was fabricated based on the modification of a patterned ITO electrode using chitosan (CS), modified reduced graphene oxide (rGO) and nanocomposite (CS–rGO). When CS–rGO was drop-coated onto an ITO electrode, the resulting CS–rGO/ITO had abundant amine groups and a biocompatible environment. The immunorecognitive interface was then constructed by the covalent immobilization of a specific polyclonal antibody via a facile glutaraldehyde cross-linking method followed by blocking non-specific sites using bovine serum albumin (BSA). The good biocompatibility of CS combined with the excellent electron transfer performance of rGO, made it possible for the as-prepared immunosensor to realize the sensitive detection of PSA with excellent selectivity, good reproducibility and high storage stability. The determination of PSA in human serum samples was also achieved. This work demonstrates a new strategy for constructing the costless and disposable immunosensor for the reliable determination of tumor biomarkers.

## 2. Results and Discussion

### 2.1. Facile Fabrication of Disposable and Label-Free Amperometric Immunosensor

**Figure 1:**
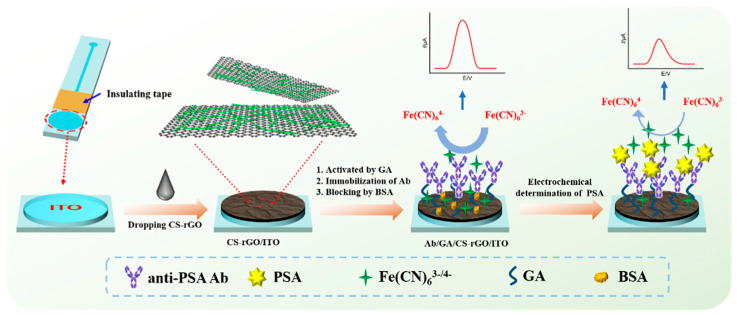
Schematic illustration of the facile fabrication of the immunosensor and amperometric determination of PSA.

A disposable sensor is crucial for making low-cost medical instruments, so the development of a low-cost, disposable, high-performance electrode for tumor marker detection is of great significance. As demonstrated in Figure 1, patterned indium tin oxide (ITO) with a circular electrode area and linear ITO wire was employed as the supporting disposable electrode in this work. Insulating tape was pasted at the junction of the circular and linear structures. Thus, the circular structure was used as the active area of the electrode, ensuring that area of each electrode was consistent. ITO is an n-type semiconductor material based on tin-doped indium oxide, which has excellent electrical conductivity (resistivity of 1–3 × 10^−4^ Ω·cm) and is currently the most widely used transparent conductive oxide material. Using ITO as the working electrode to construct a disposable electrochemical immunosensor has the advantages of low fabrication cost, easy patterning, and short pretreatment time.

To further increase the active area of the electrode and provide a biocompatible surface for constructing the immunorecognitive interface, a chitosan (CS)-modified reduced graphene oxide (rGO) nanocomposite (CS–rGO) was used to modify the ITO electrode. As is known, chitosan is currently the second largest renewable resource, and its content in nature is second only to cellulose [34,35,36]. As the product of the acetylation of chitin, CS is the only known basic polysaccharide. Owing to the large number of amino and hydroxy groups in its structure, CS has good film-forming properties, good biocompatibility and high chemical reactivity. However, the poor mechanical property of CS film and its easy hydrolysis/swelling in water severely limit its application. It has been proven that graphene materials can effectively improve the strength, stability and water resistance of polymers [37,38]. CS–rGO can easily be synthesized by reducing GO in presence of CS. Due to the positive charge caused by the protonation of amino groups, CS can electrostatically interact with GO, which is negatively charged due to the ionization of oxygen-containing groups. The formation of CS–rGO in this manner also avoids agglomeration between graphene sheets. Glutaraldehyde (GA) is then used as a bifunctional reagent to link to the CS amino group and introduce an aldehyde group (GA/CS–rGO/ITO) for covalent reaction with the amino groups in the antibody to achieve covalent immobilization of the anti-PSA antibody (Ab). Subsequently, non-specific sites on the electrode surface were blocked using BSA to obtain the immunosensor (Ab/GA/CS–rGO/ITO). The fabrication of the immunosensor has the advantages of simple process and easy operation. To realize the electrochemical detection of PSA, Fe(CN)_6_^3−/4−^ in solution was used as an electrochemical probe. When PSA specifically binds to antibodies at the immune recognitive interface, the antigen–antibody complex is formed on the electrode surface. The large size and high resistance of the immunocomplex hinders electron exchange between Fe(CN)_6_^3−/4−^ and the supporting electrode, resulting in a significant decrease in the electrochemical signal. Based on this mechanism, sensitive amperometric detection of PSA can be achieved.

**Figure 2:**
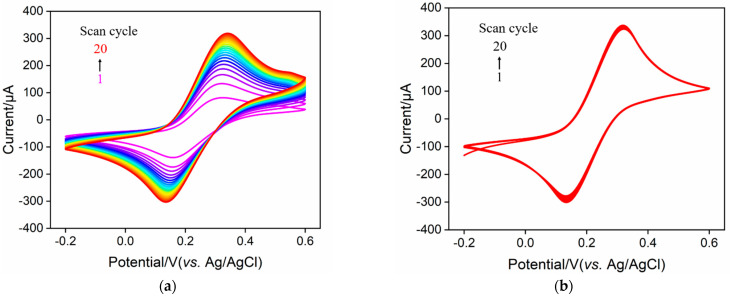
CV curves obtained on CS/ITO (**a**) or CS-rGO/ITO (**b**) electrode for 20 consecutive scans. The electrolyte solution is Fe(CN)_6_^3−/4−^ (2.5 mM) containing 0.1 M KCl.

The effect of the mass ratio of CS to rGO was investigated by changing the mass ratio between the original CS and GO. Briefly, three mass ratios of CS and GO (5.5, 11.0 and 22.0) were used for the synthesis of CS–rGO. The resulting three solutions were left to stand for 2 h. When the used mass ratio was small (5.5 or 11.0), obvious precipitation appeared at the bottom of the solution, indicating that the CS–rGO was easy to agglomerate when there was less CS as the protective agent. In contrast, there was no precipitation in the synthesized nanocomposite solutions at a high CS to GO ratio (22.0), demonstrating the stable dispersion of the CS–rGO nanocomposite. Thus, this mass ratio was used to synthesize CS–rGO nanocomposites in subsequent experiments. Figure 2 shows the cyclic voltammetry (CV) curves of CS/ITO and CS–rGO/ITO electrodes in Fe(CN)_6_^3−/4−^ solution for 20 consecutive scans. It can be seen that the CS–rGO-modified electrode had a significantly improved electrochemical signal compared with the CS-modified electrode, which was attributed to the high electron transfer rate of rGO. More noteworthy was that the peak current obtained on the CS/ITO remarkably increased during the continuous scanning, indicating the swelling and shedding of CS film. In contrast, CS–rGO/ITO showed good stability with no significant change in the peak current after 20 consecutive scans. This phenomenon illustrated that the rGO composite significantly improved the stability of CS films. rGO might act as an enhancer to reinforce CS composite film at the electrode surface. Thus, CS–rGO with a rGO-crosslinked structure forms a stable film on the ITO surface (CS–rGO/ITO) and provides a biocompatible environment with chemical reactivity for the further covalent antibody immobilization.

### 2.2. Characterization of CS–rGO Nonocomposite

**Figure 3:**
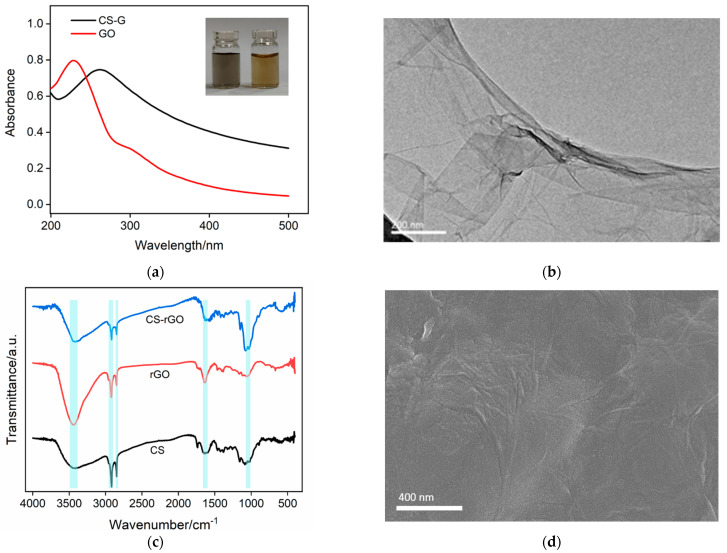
(**a**) UV-Vis absorption spectrum of GO and CS-rGO. Insets are photographs of rGO (left) and GO (right) solutions. (**b**) TEM image of CS-rGO. (**c**) FT-IR spectra of CS, rGO synthesized in absence of CS and CS-rGO. (**d**) SEM image of CS-rGO/ITO.

As shown the inset in Figure 3a, the brown GO solution (right panel) was employed as the raw material for the synthesis of CS–rGO. When GO was reduced in the presence of CS, the CS–rGO solution turned black (left panel). The structural changes of the two solutions were characterized by UV-Vis spectroscopy. Figure 3a presents the UV-Vis absorption spectra of GO and CS–rGO. It can be seen that GO had characteristic absorption peaks at 230 and 300 nm, corresponding to the π–π* transition of the conjugated C-C=C (sp^2^ C) and the n-π* transition of C=O, respectively. For CS–rGO, the absorption peak at 230 nm red-shifted to 268 nm, indicating that the reduction of GO by hydrazine hydrate led to the restoration of the electron-conjugated structure within the graphene sheet. In addition, the absorption peak at 300 nm becames indistinct, indicating that part of the oxygen-containing groups was reduced. These results demonstrate the successful synthesis of rGO through the reduction of GO. Figure 3b gives the TEM image of CS–rGO, and a single-layered graphene sheet structure was revealed.

The CS composite with rGO was also investigated using Fourier transform infrared spectroscopy (FT-IR). Figure 3c shows the FT-IR spectra of CS, rGO synthesized without the protection of CS, and the CS–rGO nanocomposite. The characteristic peaks of CS included a broadened absorption peak at ~3340 cm^−1^ where the stretching vibration of the hydroxyl group (-OH) overlapped that of the amino group (-NH_2_). The peaks at 2918 and 2849 cm^−1^ corresponded to the stretching vibration peaks of C–H, and the peak at 1640 cm^−1^ was the stretching vibration signal of acetylated N–H, indicating the incomplete deacetylation of chitin. In absence of CS, rGO can also be synthesized using the same procedure. However, it has poor water dispersibility and agglomerates easily. Four characteristic peaks were observed in its spectrum including the stretching vibration of C=O at 1736 cm^−1^, the stretching vibration peaks of C=C at 1639 cm^−1.^ and C–O–C at 1058 cm^−1^, and the vibrational peak of the -OH groups at 3340 cm^−1^. These characteristic peaks demonstrated that conjugated C=C structure and some oxygen-containing groups are both in rGO. In the case of CS–rGO, all characteristic peaks of CS and rGO appear, proving it is an effective composite. Figure 3d is the scanning electron microscope (SEM) image of the CS–rGO-modified ITO electrode. As can be seen, the CS–rGO film was relatively flat although some wrinkles were observed, possibly because of the lamellar structure of rGO.

### 2.3. Characterization of Immuonsensor Fabrication

**Figure 4:**
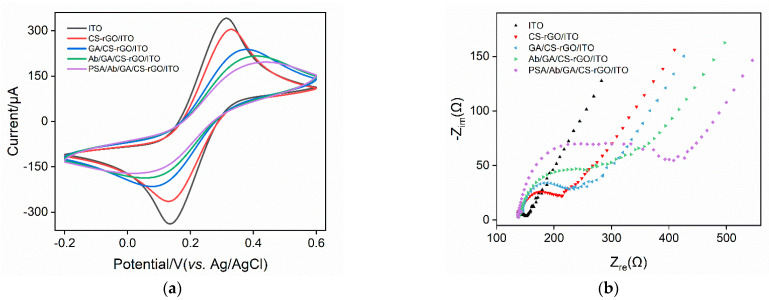
Cyclic voltammetry (**a**) with EIS curves (**b**) from different electrodes. The electrolyte solution was Fe(CN)_6_^3−/4−^ (2.5 mM) containing 0.1 M KCl.

The feasibility of constructing an immunosensor was investigated by examining the signals of a standard redox probe on each electrode obtained during the preparation process. Figure 4a shows the cyclic voltammetry (CV) curves from on different electrodes in the Fe(CN)_6_^3^^−/4^^−^ solution. As shown, Fe(CN)_6_^3^^−/4^^−^ has a pair of reversible redox peaks on the supporting ITO electrode. When the CS-G layer was modified on the ITO surface, the peak current of the redox probe decreased, accompanied by a slight increase of the peak-to-peak potential difference. This was attributed to the poor electrical conductivity of CS as a polymeric material. As CS contains a large number of amino groups, it can react with the bifunctional reagent glutaraldehyde to generate a surface with aldehyde groups. During this process, part of the CS amino groups becomes cross-linked by glutaraldehyde, resulting in a further decrease in the peak current of the redox probe on the GA/CS-G/ITO electrode and a further increase in the peak-to-peak potential difference. Subsequently, the amino group in the antibody reacts with the aldehyde group on the electrode surface to realize the covalent immobilization of the antibody. After the non-specific sites on the electrode are blocked using BSA, the prepared immunosensor is designated as an Ab/GA/CS-G/ITO electrode. Owing to the non-conductive nature of the immobilized antibody and the blocked BSA, the immunosensor had a small peak current and a large peak-to-peak potential difference. After the immunosensor was incubated with PSA, the redox probe signal was further reduced, proving the formation of the antigen–antibody complex through specific recognition. The above results demonstrated the efficient preparation of the immunosensor and the specific recognition of PSA by the immobilized antibody.

Electrochemical impedance spectroscopy (EIS) has also been used to study changes in the electrode interface during immunosensor construction. Figure 4b shows the plot curves for different electrodes. Each curve consists of a semicircle in the high-frequency region, representing the electron transfer process. The linear part in the low-frequency region corresponds to the diffusion process. As known, the equivalent diameter of the semicircle in the high frequency region is the apparent charge transfer resistance (*R*_ct_). As shown, the *R*_ct_ of the electrode increased when the ITO was modified by CS–rGO, which was attributed to low CS conductivity. The subsequent cross-linking of GA further increased the *R*_ct_ of the electrode. After the electrode surface was further modified with Ab and blocked with BSA, the increased *R*_ct_ indicated the increase in surface resistance due to the further binding of protein layers. When the immune electrode combined with PSA, the formation of the antigen–antibody complexes significantly increased the *R*_ct_, suggesting the effectiveness of the immunorecognitive interface.

### 2.4. Optimization of Conditions for Immuonsensor Fabrication

**Figure 5:**
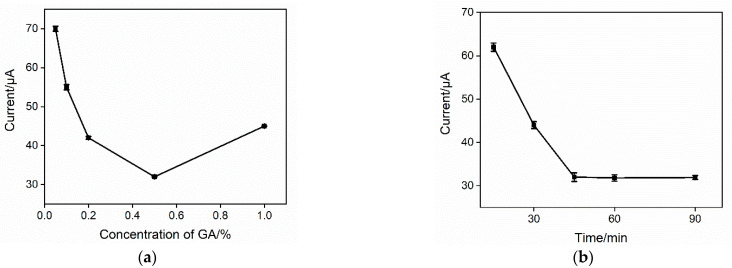
DPV peak current for the PSA-bound immunosensor prepared using different concentrations of GA (**a**) or different reaction times for the immobilization of the antibody (**b**). The electrolyte is Fe(CN)_6_^3^^−/4^^−^ (2.5 mM) containing 0.1 M KCl. The concentration of PSA was 1 ng mL^−1^. Error bars represent the standard deviation of three measurements.

To obtain the best detection sensitivity, the GA concentration for the preparation of the immunosensor and the reaction time to immobilize the antibody were optimized. The electrochemical signal of Fe(CN)_6_^3^^−/4^^−^ on the immunosensor was measured after the binding of PSA. As shown in Figure 5a, when GA concentration was low, the increase in the GA concentration resulted in a decreased redox probe signal, indicating the increased amount of the bound PSA on the fabricated immunosensor. However, when the concentration of GA increased further, the redox probe signal on the PSA-bound immunosensor first decreased then increased. When the concentration of GA was too high, the two aldehyde groups in the GA molecule would be cross-linked with the amino groups in the chitosan structure, leading to a decreased immobilized antibody. Thus, 0.5% GA was chosen for further investigation. As revealed in Figure 5b, the electrochemical signal of the PSA-bound electrode was almost unchanged when the reaction time between the antibody and the aldehyde-containing surface was 45 min. Thus, this time was chosen to fabricate the immunosensor.

### 2.5. Sensitive Amperometric Determination of PSA

**Figure 6:**
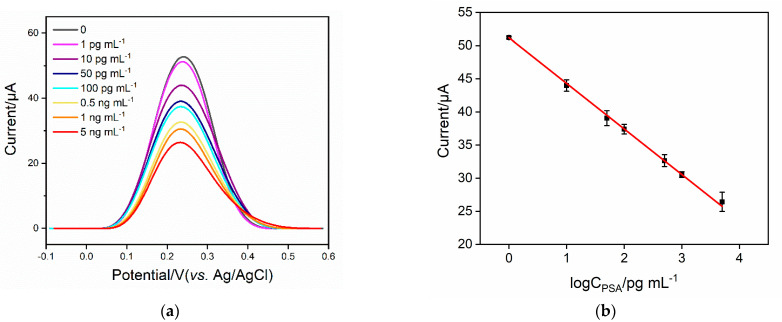
(**a**) Differential pulse voltametric curves of the Ab/GA/CS-rGO/ITO electrode towards various concentrations of PSA. (**b**) The corresponding calibration curves to determine PSA using the Ab/GA/CS-rGO/ITO electrode. Error bars represent the standard deviation of three measurements.

The performance of the developed immunosensor for determining PSA was investigated. Figure 6a displays DPV curves from an Ab/GA/CS–rGO/ITO electrode in the presence of different concentration of PSA. As shown, the peak current gradually decreased when the concentration of PSA increased. A linear correlation was revealed between the peak current (*I*, μA) of the redox probe and the logarithm of the concentration of PSA (log*C*_PSA_) in the range from 1 pg mL^−1^ to 5 ng mL^−1^ (*I* = −6.88 *C* +51.2, *R*^2^ = 0.998) (Figure 6b). The limit of detection (LOD) was 0.8 pg mL^−1^ at a signal-to-noise ratio of 3.

### 2.6. Selectivity, Reproducibility, and Stability of the Developed Immunosensor

**Figure 7:**
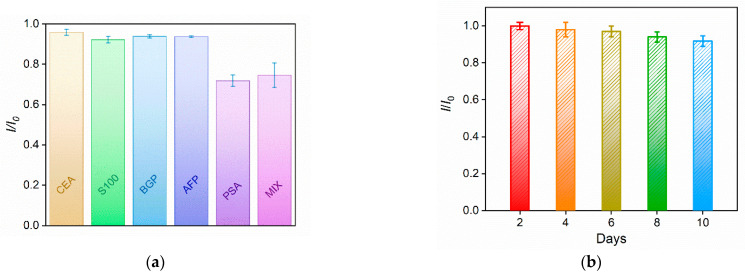
(**a**) Relative ratio of peak current before (*I*_0_) and after (*I*) incubation with different proteins or their mixture c. The concentration of PSA and other proteins was 1 and 10 ng mL^−1^, respectively. Error bars represent the standard deviation of three measurements. (**b**) Relative ratio of peak currents before (*I*_0_) and after (*I*) storing the immunosensor at 4 °C for different times. The peak currents were obtained after the immunosensors bound with 1 ng mL^−1^ of CEA. Error bars represent the standard deviation of three measurements.

To investigate the selectivity of the constructed immunosensor, it was incubated with PSA or other tumor markers including carcinoembryonic antigen (CEA), S100 calcium-binding protein β (S100), bone gamma-carboxyglutamate protein (BGP), alpha-fetoprotein (AFP), or a mixture. The peak current of the redox probe on the immunosensor before and after binding with one or a mixture of the above proteins was measured and the results are shown in Figure 7a. As shown, only PSA or a mixture of all proteins significantly changed the Fe(CN)_6_^3^^−/4^^−^ signal. No remarkable changes in the electrochemical signal were observed in the presence of one of the other proteins, indicating the specific binding of PSA on the immunorecognitive interface and the high selectivity of the immunosensor. The inter-electrode reproductivity and storage stability of the immunosensor were also studied. The reproducibility of the immunosensor electrodes was evaluated by preparing five electrodes using the same procedure in a batch. The RSD for detecting PSA was 2.1%. When the immunosensor was stored at 4 °C, it retained 91% of the original signal after 10 days, suggesting high storage stability (Figure 7b).

### 2.7. Determination of PSA in Human Serum

The feasibility of the developed immunosensor for practical application was evaluated by measuring the PSA concentration in the serum of a healthy human male. The PSA concentration that was determined using the proposed immunosensor (1.26 ng mL^−1^) was in agreement with that obtained by the enzyme-linked immunosorbent assay (ELISA) electrochemiluminescence (ECL) analyzer (1.33 ng mL^−1^). In addition, different concentrations of standard PSA solutions were artificially added to the human serum samples to simulate cancer patients with different PSA concentrations. In this standard addition method, PSA recoveries ranged from 97.4 to 102%, showing good accuracy (Table 1). The results indicated the potential for the immunosensor for the clinical analysis of PSA.

## 3. Materials and Methods

### 3.1. Chemicals and Materials

Monolayered graphene oxide (GO) aqueous dispersion (1 mg/g) was purchased from Hangzhou GaoxiTech (Hangzhou, China). Hydrazine hydrate and concentrated hydrochloric acid (HCl) were purchased from Hangzhou Shuanglin Chemical Reagent Co., Ltd. (Hangzhou, China). Chitosan (CS), sodium dihydrogen phosphate (NaH_2_PO_4_·2H_2_O), disodium hydrogen phosphate (Na_2_HPO_4_·12H_2_O), glutaraldehyde (GA), potassium chloride (KCl), potassium ferricyanide (K_3_[Fe(CN)_6_]) and potassium ferrocyanide (K_4_[Fe(CN)_6_]) were purchased from Shanghai Aladdin Biochemical Technology Co., Ltd. (Shanghai, China). Phosphate buffer solution (PBS) is prepared by mixing Na_2_HPO_4_·12H_2_O and NaH_2_PO_4_·2H_2_O in a certain proportion. Prostate specific antigen (PSA), monoclonal anti-PSA antibody (Ab), carcinoembryonic antigen (CEA), bone gamma-carboxyglutamate protein (BGP), and alpha-fetoprotein (AFP) were purchased from Beijing Keyue Zhongkai Biotech Co., Ltd. (Beijing, China). S100 calcium-binding protein β (S100) was purchased from Proteintech Co., Ltd. (Wuhan, China). All chemicals used in this experiment were of analytical grade and used without further treatment. The deionized water (18.2 MΩ•cm) used in the experiment was prepared by Mill-Q Systems (Millipore Company, Burlington, MA, USA). Indium tin oxide conductive glass (ITO, square resistance <17 Ω/sq, thickness of ITO layer: 100 ± 20 nm) was purchased from Zhuhai Kaiwei Optoelectronics Technology Co., Ltd. (Shanghai, China).

### 3.2. Experiments and Instrumentations

The morphological structure of CS–rGO was collected on a SU8010 (Hitachi, Japan) field emission microscope (SEM) with an accelerating voltage of 5.0 kV. Ultraviolet-visible (UV) absorption spectroscopy was performed on a UV-2450 spectrometer (Shimadzu, Japan). Cyclic voltammetry (CV) measurement was performed on a CHI660D electrochemical workstation (Shanghai Chenhua Co., Ltd., Shanghai, China). Electrochemical impedance spectroscopy (EIS) and differential pulse voltammetry (DPV) measurements were performed on an Autolab (PGSTAT302N) electrochemical workstation (Metrohm, Switzerland). The step potential, pulse amplitude, pulse time and interval time were 0.005 V, 0.05 V, 0.05 s, and 0.2 s, respectively. A conventional three-electrode system was used in all electrochemical measurements. Briefly, bare or modified ITO was used as the working electrode; a platinum wire electrode was used as the counter electrode; and an Ag/AgCl electrode (saturated KCl) was the reference electrode. Fourier transform infrared spectroscopy (FT-IR) was measured using a Vertex 70 spectrometer (Bruker, USA) through the KBr tablet method.

### 3.3. Synthesis of CS–rGO

As previously reported in [39], 4 mL of GO (1 mg mL^−1^) was slowly added to 36 mL of chitosan aqueous solution (0.25 wt%, pH = 3) with stirring until the solution became transparent. After sonication for 10 min, the solution was transferred to a round-bottomed flask and heated to 80 °C. After hydrazine hydrate (50 wt%, 20 μL) was added under vigorous stirring, the solution reacted at 80 °C for 3 h. The resulting solution was centrifuged at 3000 rpm to remove large material agglomerated during the reduction process. Then, the supernatant was further centrifuged at 15,000 rpm for 10 min to obtain CS–rGO solids. After being washed with 0.1 mM HCl three times, the CS–rGO was freeze-dried for use.

### 3.4. Fabrication of the Immunosensor

Before use, the ITO electrode (2.5 × 5.0 cm; active electrode area, 0.25 cm^2^) was soaked with 1 M NaOH for 2 h. After being washed with deionized water, it was successively sonicated in acetone, ethanol, and deionized water for 5 min; then,10 μL of CS–rGO (0.25 mg mL^−1^) was dropped on the ITO electrode. After drying at room temperature, CS–rGO-modified electrode was obtained (CS–rGO/ITO). To prepare the immunorecognitive interface, the CS–rGO/ITO electrode was first soaked in GA solution (0.5 wt%) to introduce the aldehyde groups. After reacting in the dark at 37 °C for 20 min, the electrode was thoroughly washed with deionized water to remove excess GA. The resulting electrode was denoted as GA/CS–rGO/ITO. Finally, the GA/CS–rGO/ITO electrode was incubated in 100 μg mL^−1^ of PSA antibody at 37 °C for 60 min, and the electrode was thoroughly rinsed with PBS (0.01 M, pH = 7) to remove unbound antibodies. The resulting electrode was immersed in BSA solution (1%) for 60 min to block non-specific sites. After washing away unbound BSA with PBS (0.01 M, pH = 7), the immunosensing electrode was denoted as Ab/GA/CS–rGO/ITO.

### 3.5. Electrochemical Determination of PSA

To detect PSA, the Ab/GA/CS–RGO/ITO electrode was incubated with different concentrations of PSA at 37 °C for 45 min. It was then carefully washed with PBS (0.01 M, pH = 7) to remove unbound PSA. Then, differential pulse voltammetry (DPV) was used to measure the electrochemical signals of PSA-bound electrodes in an Fe(CN)_6_^3−^/Fe(CN)_6_^4−^ solution (2.5 mM, containing 0.1 M KCl). In the analysis of real sample, human serum was first diluted with PBS (0.01 M, pH = 7) by a factor of 100 and then determined. Electrochemical signals were then measured before and after the immunosensor was incubated in the diluted serum.

## 4. Conclusions

In summary, a disposable amperometric immunosensor was fabricated for label-free electrochemical determination of prostate specific antigen based on chitosan-functionalized reduced graphene oxide nanocomposite. The construction was simple and easy to operate. The modified CS–rGO nanocomposite offered high biocompatibility and reactive functional groups for covalent antibody immobilization. At the same time, rGO provided excellent electron transfer performance. The fabricated immunosensor realized the specific binding of PSA with high sensitivity and good stability. In addition, the supporting ITO electrode was used as a disposable electrode with the advantages of low cost (no more than CNY5 per electrode) and easy patterning. In addition to glass substrates, ITO film can also be prepared on different substrate materials including polymers (e.g., cellulose, polymethyl methacrylate (PMMA), polyethylene terephthalate (PET), polyethylene naphthalate (PEN), acrylic (AC), polycarbonate (PC) and silicon (100), indicating great potential for wearable devices.

## Figures and Tables

**Table 1:** Determination of PSA in human serum.

Sample	Spiked (ng mL^−1^)	Found (ng mL^−1^)	RSD (%)	Recovery (%)
Human serum ^a^	1.00	1.01	2.8	101
	10.0	9.74	1.9	97.4
	100	102	3.1	102

^a^ Samples were diluted 100 times. The concentration indicated in the table was the concentration before dilution.

## Data Availability

The data presented in this study are available on request from the corresponding author.
